# Synovial microparticles from arthritic patients modulate chemokine and cytokine release by synoviocytes

**DOI:** 10.1186/ar1706

**Published:** 2005-03-01

**Authors:** René J Berckmans, Rienk Nieuwland, Maarten C Kraan, Marianne CL Schaap, Desirée Pots, Tom JM Smeets, Augueste Sturk, Paul P Tak

**Affiliations:** 1 Department of Clinical Chemistry, Academic Medical Center, University of Amsterdam, Amsterdam, The Netherlands; 2 Department of Clinical Immunology and Rheumatology, Academic Medical Center, University of Amsterdam, Amsterdam, The Netherlands

## Abstract

Synovial fluid from patients with various arthritides contains procoagulant, cell-derived microparticles. Here we studied whether synovial microparticles modulate the release of chemokines and cytokines by fibroblast-like synoviocytes (FLS). Microparticles, isolated from the synovial fluid of rheumatoid arthritis (RA) and arthritis control (AC) patients (*n *= 8 and *n *= 3, respectively), were identified and quantified by flow cytometry. Simultaneously, arthroscopically guided synovial biopsies were taken from the same knee joint as the synovial fluid. FLS were isolated, cultured, and incubated for 24 hours in the absence or presence of autologous microparticles. Subsequently, cell-free culture supernatants were collected and concentrations of monocyte chemoattractant protein-1 (MCP-1), IL-6, IL-8, granulocyte/macrophage colony-stimulating factor (GM-CSF), vascular endothelial growth factor (VEGF) and intracellular adhesion molecule-1 (ICAM-1) were determined. Results were consistent with previous observations: synovial fluid from all RA as well as AC patients contained microparticles of monocytic and granulocytic origin. Incubation with autologous microparticles increased the levels of MCP-1, IL-8 and RANTES in 6 of 11 cultures of FLS, and IL-6, ICAM-1 and VEGF in 10 cultures. Total numbers of microparticles were correlated with the IL-8 (*r *= 0.91, *P *< 0.0001) and MCP-1 concentrations (*r *= 0.81, *P *< 0.0001), as did the numbers of granulocyte-derived microparticles (*r *= 0.89, *P *< 0.0001 and *r *= 0.93, *P *< 0.0001, respectively). In contrast, GM-CSF levels were decreased. These results demonstrate that microparticles might modulate the release of chemokines and cytokines by FLS and might therefore have a function in synovial inflammation and angiogenesis.

## Introduction

Cell-derived microparticles, predominantly from platelets and erythrocytes, are present in human blood. The presence of such microparticles has been associated with the activation of coagulation [1-3]. We demonstrated recently that synovial fluid from the inflamed joints of rheumatoid arthritis (RA) and arthritis control (AC) patients also contains cell-derived microparticles. These microparticles originate from monocytes and granulocytes, and to a smaller extent from lymphocytes [4]. Synovial microparticles are strongly procoagulant via an initiation mechanism dependent on tissue factor and factor VII(a). We therefore proposed that such microparticles might contribute to the local formation of fibrin clots, the so-called rice bodies.

Fibroblast-like synoviocytes (FLS) have a key function in the development of sustained inflammation and angiogenesis in arthritic joints [5-8]. On activation *in vitro *by cytokines or bacterial lipopolysaccharides, FLS produce chemokines including monocyte chemoattractant protein-1 (MCP-1) [9,10], IL-8 [11-13] and RANTES [11,14], cytokines such as IL-6 [12,13] and granulocyte/macrophage colony-stimulating factor (GM-CSF) [13,15,16], and angiogenic factors such as vascular endothelial growth factor (VEGF) [17,18].

The presence of leukocyte-derived microparticles in blood has been associated with systemic inflammatory disorders, such as pre-eclampsia [19], sepsis with multiple organ failure [20], and meningococcal septic shock [21], and leukocyte-derived microparticles – but not platelet-derived microparticles – trigger the expression of IL-6 and MCP-1 by endothelial cells [22,23]. However, it is unknown whether leukocytic microparticles contribute to local inflammation. We therefore determined whether isolated synovial microparticles of arthritis patients trigger the release of (pro-) inflammatory and angiogenic mediators by cultured autologous FLS from inflamed joints of RA and AC patients.

## Materials and methods

### Patients

Paired synovial fluid, plasma and synovial tissue specimens were collected from eight RA and three undifferentiated AC patients. The diagnosis of AC patients stayed unchanged during 1 year of follow-up. The RA patients fulfilled the criteria of the 1987 Criteria of the American College of Rheumatology. The study was approved by the Medical Ethical Committee of the Academical Medical Center of the University of Amsterdam, and informed consent was obtained to participate in the present study. The demographic and clinical data are summarized in Table 1.

### Reagents and assays

Anti-CD4 labeled with phycoerythrin (PE; CLB-T4/2 6D10, IgG_1_) and anti-CD66e-PE (CLB-gran/10 IH4Fc, IgG_1_) were obtained from the Central Laboratory of the Netherlands Red Cross Blood Transfusion Service (CLB; Amsterdam, The Netherlands), anti-glycophorin A-PE (JC159, IgG_1_) was from DakoCytomation (Glostrup, Denmark). Anti-CD8-PE (Leu™-2a, IgG_1_), anti-CD14-PE (MφP9, IgG_2b_), anti-CD20-PE (L27, IgG_1_), anti-CD61-PE (VI-PL2, IgG_1_) and IgG_1_-PE (X40) were from Becton Dickinson (BD, San Jose, CA, USA), and anti-IgG_2b_-PE (MCG2b) was from Immuno Quality Products (Groningen, The Netherlands). IL-6, IL-8 and intracellular adhesion molecule-1 (ICAM-1; Diaclone Research, Besançon, France) and MCP-1, RANTES, VEGF and GM-CSF (BioSource International, Camarillo, CA, USA) were determined by ELISA. IL-1β was obtained from Roche Diagnostics (Mannheim, Germany)

### Collection of the synovial biopsy and culture of FLS

Synovial tissue was collected from an actively inflamed joint by small-needle arthroscopy under local anesthesia with a 2.5 mm biopsy forceps to sample from different areas throughout the knee joint [24]. Synovial tissue was placed in Dulbecco's modified Eagle's medium (Life Technologies, Paisley, Renfrewshire, UK) supplemented with 10% FCS, 50 μg/ml streptomycin, 50 IU/ml penicillin and 2 mM L-glutamine and subjected to tissue digestion within 2 hours, as described previously [25]. The cells were cultured at 37°C and 5% CO_2_. After the second passage, FLS were seeded into 24-well flat-bottomed plates (Costar, Acton, MA) and maintained for 24 hours in culture medium containing 1% FCS.

### Collection of synovial fluid and blood samples

Immediately before the arthroscopy, we collected synovial fluid (4.5 ml) from the same joint and also venous blood (4.5 ml) into tubes containing 0.5 ml of 3.2% sodium citrate (BD). Immediately after collection, a further 0.5 ml of 3.2% sodium citrate was added to the synovial fluid to prevent clotting. Cells were removed from both blood and synovial fluid by centrifugation for 20 min at 1,550 *g *and 20°C. For all determinations, aliquots of cell-free plasma and synovial fluid were snap-frozen in liquid nitrogen for at least 15 min and stored at -80°C until use.

### Microparticle isolation

For flow-cytometric analysis, cell-free synovial fluid aliquots (250 μl) were thawed on melting ice and centrifuged for 30 min at 17,570 *g *and 20°C to pellet the microparticles. Supernatant (225 μl) was removed and microparticles were resuspended in 225 μl PBS (154 mM NaCl, 1.4 mM phosphate, pH 7.4), containing 10.9 mM trisodium citrate. After centrifugation for 30 min, supernatant (225 μl) was again removed and microparticles were resuspended in 150 μl of PBS/citrate buffer. For the FLS experiments, microparticles were isolated from 1 ml of synovial fluid by centrifugation for 1 hour at 17,570 *g *and 20°C. Supernatant (975 μl) was removed and replaced by 975 μl of PBS containing trisodium citrate. Microparticles were resuspended and again pelleted by centrifugation for 1 hour at 17,570 *g *and 20°C. Again, 975 μl of supernatant was removed and microparticles were resuspended in the remaining 25 μl. This microparticle suspension was added to a final volume of 1 ml of culture medium in which FLS had been maintained for 24 hours. Where indicated, a higher concentration of microparticles was also tested for its ability to activate FLS when sufficient synovial fluid was available. These microparticles, isolated from 3 ml of synovial fluid, were also concentrated into 25 μl of PBS containing trisodium citrate. Microparticle suspensions were each added to FLS cultures from the same donor to mimic the situation *in vivo *as much as possible.

### Incubation of FLS with microparticles

FLS were quiescent after incubation for 24 hours in medium containing 1% FCS. After 24 hours, this medium (1 ml) was replaced by culture medium containing 1% FCS without any other addition (1 ml; control), or by 975 μl of culture medium plus (1) 25 μl of IL-1β (125 pg/ml final concentration), (2) 25 μl of microparticle suspension or (3) 25 μl of microparticle-free synovial fluid that had been diluted 1:9 in PBS (that is, containing 2.5 μl of the original synovial fluid; this quantity was chosen arbitrarily to correct for both the onefold (unconcentrated) and threefold concentrated microparticle suspensions that, after washing of the microparticles, still contained about 0.7 and 2.1 μl of synovial fluid, respectively). Because individual FLS cultures showed a considerable variation in (mediator) response to the positive control, namely IL-1β, we expressed the response of each FLS culture to microparticles as a percentage of the IL-1β-induced response.

### Flow-cytometric analysis

Microparticles were measured by flow cytometry with a method that differed slightly from that used previously [4]. In the present study, the microparticles were not washed by centrifugation after being labeled with antibodies because this resulted in a selective loss of microparticle populations. In brief, 5 μl of the microparticle suspension was added to a mixture of PBS (45 μl) containing 2.5 mM CaCl_2 _and 5 μl of PE-labeled mAb, and incubated for 15 min in the dark at ambient temperature (20 to 22°C). The following (final concentrations) of mAbs were used: anti-CD4-PE (0.5 μg/ml), anti-CD8-PE (0.25 μg/ml), anti-CD14-PE (0.25 μg/ml), anti-CD20-PE (0.5 μg/ml), anti-CD61-PE (0.5 μg/ml), anti-CD66e-PE (0.25 μg/ml) and anti-glycophorin A-PE (0.25 μg/ml). PE-labeled IgG_1 _and IgG_2b _(both at 0.5 μg/ml) were used as isotype-specific control antibodies. After incubation, 900 μl of PBS/CaCl_2 _was added. Samples were analyzed on a FACSCalibur (BD) and data were analyzed with CellQuest™ Pro software (version 4.02; BD). Both forward scatter and side scatter were set at logarithmic gain. Microparticles were identified by forward scatter, side scatter and binding of cell-specific mAb. The number of microparticles per liter of plasma or synovial fluid was estimated by using the number of events (*N*) of cell-specific mAb-binding microparticles after correction for control antibody binding: number/liter = *N *× (150/5) × (955/67) × (10^6^/250). The lower detection limit of the particle count was previously established as 10^7 ^microparticles per liter. In this formula, 150 (μl) is the final volume of the washed microparticle suspension, 5 (μl) is the volume of this suspension that is used for each labeling, 955 (μl) is the total volume of the microparticle suspension after labeling before fluorescence-activated cell sorting analysis, 67 (μl) is the average volume of the labeled microparticle suspension that is analyzed by the flow cytometer in 1 min, 10^6 ^is the conversion from μl to liter, and 250 (μl) is the original volume of the plasma or synovial fluid sample used for microparticle isolation.

### Statistical analysis

Data were analyzed with GraphPad Prism for Windows, release 3.02 (San Diego, CA, USA). Differences in the concentrations of chemokines, cytokines and VEGF between synovial fluid and plasma as well as in culture supernatants were analyzed with the Wilcoxon signed-rank test. Two-tailed significance levels were considered significant at *P *< 0.05. All data are presented as medians (range).

## Results

### Cellular origin of synovial microparticles

Previously, we found no differences between the numbers and cellular origin of microparticles in synovial fluid from RA and AC patients [4]. For all cell-specific antigens tested, the microparticle numbers of the three AC patients fell within the range of the RA patients, which is consistent with these earlier observations. The data in Table 2 therefore summarize the microparticle numbers for RA and AC patients together. Most microparticles originated from monocytes (CD14) and granulocytes (CD66e). Microparticles derived from platelets (CD61) and erythrocytes (glycophorin A) were below detection level (less than 10^7^/l) in synovial fluid from all patients, except in one RA patient who had a low but detectable number (1.7 × 10^7^/l) of platelet-derived microparticles. One other RA patient had a relatively high number of erythrocyte-derived microparticles (3.1 × 10^9^/l). Microparticles from CD4^+ ^cells were found in six RA patients and all AC patients. Microparticles from CD8^+ ^T cells were present in the synovial fluid of five RA patients and one AC patient. Microparticles from B cells were found in two RA patients only.

### Synovial microparticles stimulate FLS

FLS were quiescent after incubation for 24 hours in medium containing 1% FCS. The concentrations of all markers studied in the FLS culture supernatants are summarized in Table 3. In comparison with the control (unstimulated), IL-1β significantly increased the levels of all mediators tested, whereas the addition of microparticle-free synovial fluid affected especially the soluble ICAM-1 (sICAM-1) levels. This increase was due to its presence in the synovial fluid itself. Addition of microparticles to FLS significantly increased the levels of MCP-1 (*P *= 0.010), sICAM-1 (*P *= 0.010), IL-8 (*P *= 0.008), IL-6 (*P *= 0.042), VEGF (*P *= 0.001) and RANTES (*P *= 0.031). In contrast, the concentrations of GM-CSF decreased (*P *= 0.002).

In six patients (three RA and three AC patients), we also tested a threefold higher (final) concentration of synovial microparticles. In comparison with the 'onefold' concentration, levels of sICAM-1 (*P *= 0.031), IL-8 (*P *= 0.031) and IL-6 (*P *= 0.031) increased further and GM-CSF (*P *= 0.016) decreased further (Table 3). Levels of MCP-1 (*P *= 0.156), VEGF (*P *= 0.078) and RANTES (*P *= 0.062) also tended to increase further, but these differences did not reach statistical significance.

**Figure 1:**
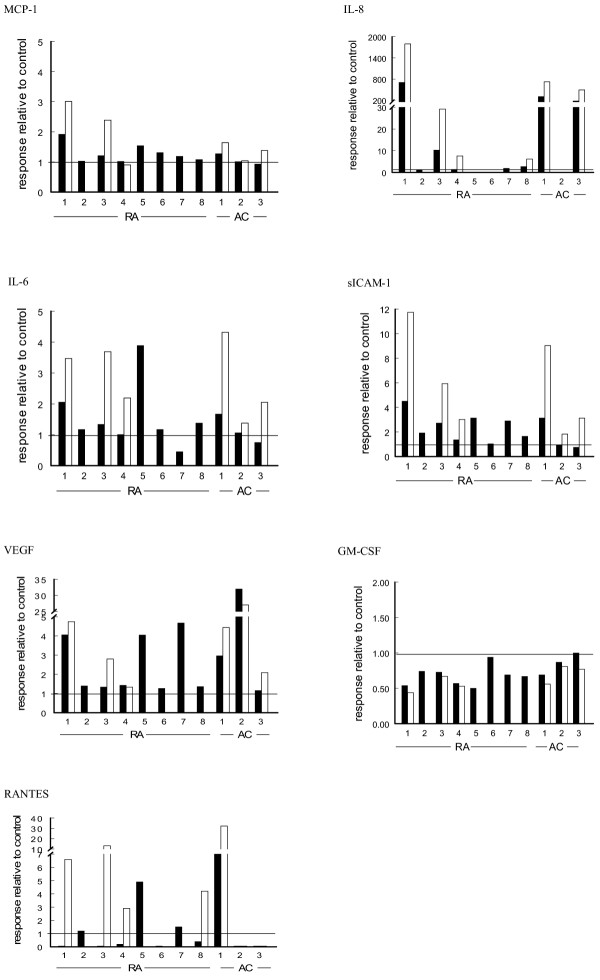
Responses of individual cultures of fibroblast-like synoviocytes from rheumatoid arthritis (RA; *n *= 8) and arthritis control (AC; *n *= 3) patients to their autologous synovial microparticles. All individual patient data for the markers studied are expressed as the concentration of the mediator in the presence of microparticles concentrated either onefold (black bars) or threefold (open bars) divided by the concentration of mediator in the presence of microparticle-free synovial fluid. ICAM-1, intracellular adhesion molecule-1; MCP-1, monocyte chemoattractant protein-1.

Because individual microparticle suspensions were tested in (autologous) FLS cultures and considerable differences were observed in the responsiveness of these individual cell cultures, the individual responses of FLS cultures are also shown (Fig. 1). The response is expressed as either an increase or a decrease relative to the control, namely the 24-hour incubation of FLS with the microparticle-free synovial fluid. Although variation between FLS cultures is apparent, the individual data substantiate the conclusions above as based on group analysis.

### Concentrations of MCP-1, IL-6, IL-8, RANTES, sICAM-1, VEGF and GM-CSF *in vivo*

**Figure 2:**
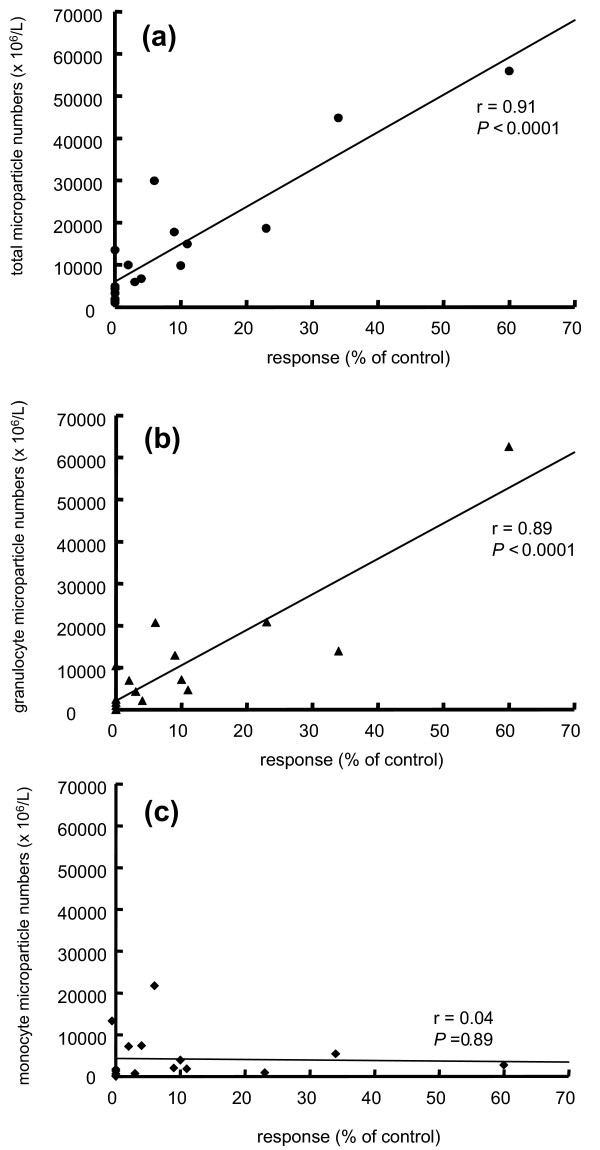
Correlation between microparticle numbers and IL-8 concentrations. Correlations are shown between IL-8 produced by FLS in response to total microparticles **(a)**, granulocyte-derived microparticles **(b) **and monocyte-derived microparticles **(c)**. Note that data obtained with FLS in response to onefold and threefold concentrated microparticle suspensions are included.

For comparison, the concentrations of the various mediators were also determined in both synovial fluid and plasma from RA and AC patients. Because only 2 values (of 36) of the AC patients fell outside the RA range, namely MCP-1 in synovial fluid and sICAM-1 in plasma from the same AC patient, all data are summarized in Table 4. In comparison with plasma, levels of MCP-1 (*P *= 0.008), IL-6 (*P *= 0.002), IL-8 (*P *= 0.002) and VEGF (*P *= 0.002) were elevated in synovial fluid, those of RANTES and ICAM-1 were decreased (*P *= 0.001 and *P *= 0.006, respectively), and GM-CSF concentrations were similar (*P *= 0.125). Figure 2 shows that both the total number of microparticles (Fig. 2a; *r *= 0.91; *P *< 0.0001) and the numbers of granulocyte-derived microparticles (Fig. 2b; *r *= 0.89, *P *< 0.0001) were correlated with the IL-8 concentrations, whereas the numbers of monocyte-derived microparticles were not (Fig. 2c; *r *= 0.04; *P *= 0.89). In addition, concentrations of MCP-1 were correlated with total numbers of microparticles (*r *= 0.81, *P *< 0.0001) and numbers of granulocyte-derived microparticles (*r *= 0.93, *P *< 0.0001), but again not with the numbers of monocyte-derived microparticles (*r *= 0.06; *P *= 0.82; data not shown). No other correlations were found between microparticle numbers and concentrations of mediators.

## Discussion

The present study shows that synovial fluid microparticles trigger FLS to release chemokines, cytokines and other mediators of inflammation. The extent to which these changes are solely induced by microparticles remains to be shown. We cannot exclude from our present data the possibility that the activation of FLS is due in part to synergistic actions of the microparticles with one or more mediators released by FLS themselves under these conditions. Neither can we exclude the possibility that microparticles activate FLS in synergy with one or more mediators already present in the synovial fluid. Nevertheless, the release of IL-8 and MCP-1 was correlated directly to both the total number of microparticles and the number of granulocyte-derived microparticles. This suggests that microparticles might trigger FLS to release these mediators. Although no correlations were found between microparticle numbers and sICAM-1, IL-6, VEGF and RANTES, a threefold increased concentration of microparticles tended to induce a higher response.

On the basis of these data it is tempting to speculate that synovial fluid microparticles promote synovial inflammation and neoangiogenesis in arthritic joints. The FLS are localized in the intimal lining layer, which directly contacts the synovial fluid compartment. Thus, synovial fluid microparticles may interact directly with the FLS, thereby modulating the release of an array of proinflammatory cytokines and chemokines. This may lead to further cell activation, neoangiogenesis and cell recruitment, constituting a proinflammatory amplification loop. Consistent with this notion is the observation that the removal of synovial fluid by arthroscopic lavage has a positive therapeutic effect in RA [26]. In addition, it has previously been shown that intra-articular injection of corticosteroids is more effective after arthrocentesis [27]. This has been explained by the effects of removal of fluid containing various proinflammatory cytokines.

At present, we can only speculate how synovial microparticles trigger FLS to produce and/or release proinflammatory mediators. Synovial microparticles originate mainly from leukocytes [4]. *In vitro*, leukocytic microparticles trigger the release of IL-6 and MCP-1 from endothelial cells [22,23]. Microparticles can contain bioactive lipids such as oxidized phospholipids, arachidonic acid and lysophosphatidic acid [28,29]. In particular, both arachidonic acid and lysophosphatidic acid are present in microparticles previously exposed to secretory phospholipase A_2 _(sPLA_2_) [30]. Arachidonic acid is transferred directly from microparticles to endothelial cells, resulting in the production of IL-6 [29]. It is unknown whether lysophosphatidic acid, a multifunctional lipid mediator that induces cell proliferation, migration and survival, is also directly transferred [31]. Synovial microparticles have been exposed to high levels of sPLA_2 _*in vivo *and are therefore likely to contain elevated levels of bioactive lipids. Thus, we propose that synovial microparticles might directly transfer bioactive lipids to FLS, thereby modulating the production and/or release of proinflammatory mediators. For this transfer, a direct interaction between microparticles and the FLS is essential. Because microparticles expose an array of cell-type-specific adhesion receptors, a direct interaction is likely. Alternatively, we cannot exclude the possibility that synovial microparticles might also contain inflammatory cytokines, because monocyte-derived microparticles generated *in vitro *were recently demonstrated to contain IL-1β [32].

Finally, the present study again showed that elevated levels of microparticles from granulocytes, monocytes and lymphocytes are present in the synovial fluid of arthritic patients. At present it is unknown why such elevated numbers of microparticles occur under these conditions. Apoptotic cells expose phosphatidylserine. Macrophages expose phosphatidylserine receptors, which efficiently initiate the recognition and subsequent removal of apoptotic cells [33,34]. It is also likely that microparticles are removed from the circulation by means of such receptors. However, synovial microparticles bind less annexin V than microparticles from plasma [4]. This decreased binding is due either to a decreased exposure of phosphatidylserine or to the presence of high levels of sPLA_2_, which competes with annexin V for binding to phosphatidylserine [35,36]. The removal of microparticles by phagocytic cells might thus be impaired in inflamed joints, resulting in the prolonged presence of microparticles and therefore in the continued stimulation of the FLS.

## Conclusion

The results of the present study suggest that microparticles modulate the release of chemokines and cytokines by FLS. However, their biological relevance, compared with or in synergy with other biological mediators in synovial fluid, remains to be determined. The beneficial effect of arthrocentesis and arthroscopic lavage in RA might be explained, at least in part, by the removal of synovial fluid microparticles.

## Abbreviations

AC = arthritis control; ELISA = enzyme-linked immunosorbent assay; FCS = fetal calf serum; FLS = fibroblast-like synoviocytes; GM-CSF = granulocyte/macrophage colony-stimulating factor; IL = interleukin; mAb = monoclonal antibody; MCP = monocyte chemoattractant protein; PBS = phosphate-buffered saline; PE = phycoerythrin; RA = rheumatoid arthritis; sICAM-1 = soluble intracellular adhesion molecule 1; sPLA_2 _= secretory phospholipase A_2_; VEGF = vascular endothelial growth factor.

## Competing interests

The author(s) declare that they have no competing interests.

## Authors' contributions

RB wrote the manuscript, guided by RN and AS, with clinical input and final correction by PT. RB, RN and AS devised the experimental design. The selection of patients and collection of synovial biopsy and blood materials were performed by MK. All experiments were performed by RB and MS except the culture of synoviocytes, which was performed by DP and TS. Supervision was fulfilled by AS and PT, with daily supervision by RN. The manuscript was read and approved by all authors.

## Figures and Tables

**Table 1:** Demographic and clinical data of the rheumatoid arthritis patients and arthritis controls

Parameter	RA patients (*n *= 8)	AC patients (*n *= 3)
Age (years)	58 (34–69)	56 (49–68)
Sex (no. of males/females)	4/4	3/0
Disease duration (months)	60 (4–360)	2 (1–12)
Rheumatoid factor	7 positive; 1 negative	1 positive; 2 negative
Tender joint count	9 (5–15)	1 (1–2)
Swollen joint count	11 (5–19)	2 (1–23)
ESR (mm/h)	46 (25–69)	38 (28–43)
Erosive disease	6 positive; 2 negative	None
No. of DMARDs	4.5 (1–5)	0
Leukocytes in SF (10^9^/l)	6.3 (4.5–7.0)	4.3 (4.2–4.5)
CRP (mg/l)	34 (8–97)	4 (<3–26)

Results are medians, with ranges in parentheses. AC, arthritis control; CRP, C-reactive protein in plasma; DMARDs, disease-modifying antirheumatic drugs; ESR, erythrocyte sedimentation rate; RA, rheumatoid arthritis; SF, synovial fluid.

**Table 2:** Microparticle numbers in synovial fluid from patients with arthritic joints

Origin	mAb	Synovial fluid
CD4^+ ^cells	CD4	191 (<10–711)
CD8^+ ^cells	CD8	<10 (<10–331)
Monocytic cells	CD14	1,315 (57–13,326)
B-cells	CD20	<10 (<10–104)
Platelets	CD61	<10 (<10–17)
Erythrocytes	Glycophorin A	<10 (<10–3,104)
Granulocytes	CD66e	2,380 (<10–20,864)

Results are medians, with ranges in parentheses. Data are the numbers (× 10^6^/l) of marker-positive microparticles from all arthritic patients (*n *= 11).

**Table 3:** Effect of synovial microparticles on the release of inflammatory mediators by fibroblast-like synoviocytes from arthritic patients (*n *= 11)

Mediator	Control		P*	MP-free synovial fluid	MP (1×)	*N*_x_/*N*_t_	*P*^†^	MP (3×)	*N*_x_/*N*_t_	*P*^‡^
									
	Unstimulated	IL-1β								
MCP-1 (pg/ml)	456	4,754	0.001	469	488	6/11	0.010	900	4/6	0.156
	(355–1,292)	(2,492–6,081)		(293–1,241)	(338–1,481)	higher		(346–2,326)	higher	
sICAM-1 (ng/ml)	0.09	0.40	0.007	1.04	2.00	8/11	0.010	6.07	6/6	0.031
	(0–0.3)	(0–0.76)		(0.34–1.84)	(0.35–4.07)	higher		(0.91–11.75)	higher	
IL-8 (pg/ml)	0	8,642	0.001	26	301	5/11	0.008	790	6/6	0.031
	(0–564)	(2,954–18,330)		(0–528)	(0–707)	higher		(0–2,100)	higher	
IL-6 (pg/ml)	74	4949	0.001	110	136	7/11	0.042	436	6/6	0.031
	(24–1,710)	(1,870–22,797)		(30–1,176)	(34–1,937)	higher		(44–3,766)	higher	
VEGF (pg/ml)	48	79	0.014	34	74	10/11	0.001	111	6/6	0.078
	(11–102)	(7–141)		(1–97)	(28–138)	higher		(27–161)	higher	
GM-CSF (pg/ml)	32	53	0.004	31	22	10/11	0.002	18	6/6	0.016
	(28–40)	(40–72)		(26–70)	(14–43)	lower		(14–25)	lower	
RANTES (pg/ml)	0	138	0.001	0	0.2	4/11	0.031	4.2	5/6	0.062
	(0–74)	(46–277)		(0–58)	(0–86)	higher		(0–32)	higher	

Results are medians, with ranges in parentheses. Concentrations of mediators were determined in the culture supernatant of the fibroblast-like synoviocytes (FLS) by ELISA as described in the Materials and methods section. FLS were incubated for 24 hours with 1 ml of culture medium containing 1% FCS (negative control), 975 μl of culture medium supplemented with either (1) 25 μl of interleukin (IL)-1β (final concentration 125 pg/ml; positive control), (2) 25 μl (onefold (1×) or threefold (3×) concentrated) microparticles (MP), or (3) MP-free synovial fluid. P*, positive versus negative control; *P*^†^, MP (1×) versus MP-free synovial fluid; *P*^‡^, MP (3×) versus MP (1×). *N*_x_/*N*_t_, number of individual culture supernatants that contained elevated or decreased concentrations of mediators after incubation for 24 hours with isolated MP compared with MP-free synovial fluid, divided by the number of patients studied. GM-CSF, granulocyte/macrophage colony-stimulating factor; sICAM-1, soluble intracellular adhesion molecule-1; MCP-1, monocyte chemoattractant protein-1; VEGF, vascular endothelial growth factor.

**Table 4:** Concentrations of inflammatory mediators in synovial fluid and plasma from arthritic patients (*n *= 11)

Mediator	Concentration		*P*
		
	Synovial fluid	Plasma	
MCP-1 (pg/ml)	134 (36–522)	34 (15–62)	0.008
sICAM-1 (ng/ml)	706 (226–1,085)	871 (657–1,691)	0.006
IL-8 (pg/ml)	614 (<50–24,630)	<50	0.002
IL-6 (pg/ml)	13,897 (35–43,131)	11 (0–57)	0.002
VEGF (pg/ml)	1,604 (528–2,506)	23 (<5–69)	0.002
RANTES (pg/ml)	7 (<5–35)	3,986 (2,920–10,037)	0.001
GM-CSF (pg/ml)	<2 (<2–39)	<2 (<2–28)	0.125

Results are medians, with ranges in parentheses. Concentrations of all mediators were determined by ELISA as described in the Materials and methods section. GM-CSF, granulocyte/macrophage colony-stimulating factor; MCP-1, monocyte chemoattractant protein-1; sICAM-1, soluble intracellular adhesion molecule-1; VEGF, vascular endothelial growth factor.
